# Microbial community composition along the digestive tract in forage- and grain-fed bison

**DOI:** 10.1186/s12917-017-1161-x

**Published:** 2017-08-17

**Authors:** Gaddy T. Bergmann

**Affiliations:** 1 Department of Ecology and Evolutionary Biology, University of Colorado, Boulder, Ramaley N122, Campus Box 334, Boulder, Colorado 80309-0334 USA; 2 Cooperative Institute for Research in Environmental Science, University of Colorado, Boulder, Box 216 UCB, Boulder, Colorado 80309-0216 USA

**Keywords:** Bison, Digestive tract, Diet, Microbiota

## Abstract

**Background:**

Diversity and composition of microbial communities was compared across the 13 major sections of the digestive tract (esophagus, reticulum, rumen, omasum, abomasum, duodenum, jejunum, ileum, cecum, ascending colon, transverse colon, descending colon, and rectum) in two captive populations of American bison (*Bison bison*), one of which was finished on forage, the other on grain.

**Results:**

Microbial diversity fell to its lowest levels in the small intestine, with *Bacteroidetes* reaching their lowest relative abundance in that region, while *Firmicutes* and *Euryarchaeota* attained their highest relative abundances there. *Gammaproteobacteria* were most abundant in the esophagus, small intestine, and colon. The forage-finished bison population exhibited higher overall levels of diversity, as well as a higher relative abundance of *Bacteroidetes* in most gut sections. The grain-finished bison population exhibited elevated levels of *Firmicutes* and *Gammaproteobacteria*. Within each population, different sections of the digestive tract exhibited divergent microbial community composition, although it was essentially the same among sections within a given region of the digestive tract. Shannon diversity was lowest in the midgut. For each section of the digestive tract, the two bison populations differed significantly in microbial community composition.

**Conclusions:**

Similarities among sections indicate that the esophagus, reticulum, rumen, omasum, and abomasum may all be considered to house the foregut microbiota; the duodenum, jejunum, and ileum may all be considered to house the small intestine or midgut microbiota; and the cecum, ascending colon, transverse colon, descending colon, and rectum may all be considered to house the hindgut microbiota. Acid from the stomach, bile from the gall bladder, digestive enzymes from the pancreas, and the relatively low retention time of the small intestine may have caused the midgut’s low microbial diversity. Differences in microbial community composition between populations may have been most strongly influenced by differences in diet (forage or grain). The clinical condition of the animals used in the present study was not evaluated, so further research is needed to establish whether the microbial profiles of some bison in this study are indeed indicative of dysbiosis, a predisposing factor to ruminal acidosis and its sequelae.

## Background

Ruminants, like all vertebrate herbivores, rely on vegetation for nourishment, but are unable to digest plant fibers without the aid of symbiotic microbes in their digestive tract [1]. Most of the ruminant digestive system is a favorable environment for microbes, as body temperature is warm and stable at around 39 °C [2]. However, the digestive tract is also a challenging environment for microbes given that they must compete for space and nutrients [3] Moreover, although ruminants provide their symbionts with abundant resources, most are in the form of cellulose, which is difficult to break down [4]. The reticulorumen and omasum are weakly acidic with a pH of 5.5–6.5, which is favorable for many microbes [5]. These sections house most of the ruminant’s symbiotic microbiota. Bacteria and protozoa are predominant, accounting for 40–60% of microbial biomass [6]. Bacteria fermenting this material release the volatile fatty acids (VFAs) acetic, propionic, and butyric acid, which the host absorbs and metabolizes [1].

As in other mammals, the ruminant gut microbiota is dominated by the bacterial phyla *Firmicutes*, *Bacteroidetes*, *Actinobacteria*, *Proteobacteria* (especially in the class *Gammaproteobacteria*) and *Verrucomicrobia* [7], while most archaea are methanogens in the phylum *Euryarchaeota* [6, 8–10]. Because the different sections of the ruminant gut present different environmental conditions, the composition of the gut microbiota changes from one section to another. The first region (a functional grouping of sections) is the foregut, which houses several common fibrolytic species [11–13], as well as amolytic, saccharolytic, lactolytic, and proteolytic species [14–17]. Second, the abomasum has a pH of 2–4, which kills and digests many of the microbes entering from the omasum, supplying the host with 60–90% of its amino acids, which are in turn absorbed in the small intestine [18–20]; [21]. Third, the small intestine is responsible for neutralizing acid from the stomach, breaking down macromolecules with enzymes, and absorbing nutrients. Microbial biomass drops sharply between the foregut and small intestine because of the acidity of the abomasum, but then increases caudally as pH rises again from 2 to 4 in the duodenum, to 4–7 in the jejunum, and finally to 7–8 in the ileum [5]. Finally, the hindgut is the last site for salt and water balance. In ruminants, this region is second to the foregut in microbial biomass. Microbiota in the cecum, colon, and rectum ferment remaining fiber and produce a variety of vitamins for their host [1, 22].

Community composition was compared along the entire digestive tract of two captive populations of plains bison (*Bison bison bison*). In domestic cattle (*Bos taurus taurus*), gut microbiota have been explored in the reticulorumen and feces [23–26], as well as across more sites along the digestive tract [5, 27]. Microbial diversity along the digestive tract has been researched in humans, rodents, and horses as well [28–30]. However, studies of the gut microbiota of bison have been limited to the reticulorumen [31–33] and feces [34]. The earlier research on ruminal microbiota predates high-throughput sequencing, so it relied on culture-dependent methods that could not detect much of the microbial diversity found in the gut. The study by [34] used culture-independent methods to characterize fecal microbiota in semi-free-ranging wood bison (*B. bison athabascae*). In the present study, two related questions were addressed. One, to what degree does microbial community composition vary along the length of the digestive tract in plains bison? Two, does the relative abundance of a given microbial taxon in a given gut section differ between two bison populations? It was hypothesized that different sections of the digestive tract would exhibit differences in microbial diversity and community composition. Likewise, for a given digestive tract section, these variables were expected to differ between the two bison populations.

## Methods

### Animals and sampling

Thirteen major sections of the alimentary canal for sampling were identified. In anterocaudal order, they were the esophagus, reticulum, rumen, omasum, abomasum, duodenum, jejunum, ileum, cecum, ascending colon, transverse colon, descending colon, and rectum. Four bison from one population were dissected on site at an abattoir on one day, and three from another population a week later, for a total of seven bison. On October 10, 2012, all four bison were grain-finished bulls, aged two-three years, from Colorado (Population B). On October 16, 2012, all three bison were forage-finished cows, aged four-fourteen years, from Nebraska (Population A). The forage-finished diet consisted of 100% roughage for the lifetime of the animals. In contrast, the grain-finished diet was 60% corn and 40% roughage from age six months, with the bison not being returned to forage prior to slaughter. For all seven individuals, double sterile cotton swabs were used to simultaneously sample the lumen and mucosa of each section in triplicate, yielding a total of 273 samples. These were transported on ice in a cooler, and stored frozen at −20 °C until processing.

### DNA isolation, amplification, and sequencing

DNA extractions were then performed on all four of the grain-fed bison, and on three of the forage-fed bison, using the MO BIO PowerSoil®-htp 96 Well Soil DNA Isolation Kit, according to the method in [35].

A portion of the 16S rRNA gene was PCR-amplified and sequenced to characterize bacterial and archaeal community composition in the bison digestive tract. To amplify these 16S rRNA genes for barcoded high throughput sequencing, the methods of [36] were followed. The primer pair 515F / 806R was used with Illumina adapters, and with a 12-bp error-correcting barcode unique to each sample on the reverse primer. The V4–V5 variable region amplified by this primer set is well-suited to accurate phylogenetic placement of bacterial sequences [37]. Together, these primers form a good “universal” primer set that amplifies nearly all bacterial and archaeal taxa with few biases [38]. Amplicons were cleaned using the MO BIO PowerClean® DNA Clean-Up Kit, and quantified using first the Quant-iT™ PicoGreen® dsDNA Assay Kit, and then the Thermo Scientific NanoDrop 1000, to determine the volume needed to produce a single composite sample with equal representation of each individual sample. The composite sample was taken to the University of Colorado Genomics Core Facility for sequencing on an Illumina MiSeq machine with the 2 × 150 bp paired-end protocol.

### Data analysis

The QIIME pipeline was used for data analysis. Quality filtering and processing of reads was performed following [39]. Only forward reads were used for downstream analyses [40]. Samples were standardized using rarefaction, and bacterial 16S rRNA sequences were clustered into operational taxonomic units (OTUs) at the 97% similarity level using the RDPII taxonomy [41]. Parametric and nonparametric statistical approaches were used to determine if communities varied across gut sections within each population of bison, as well as for a given gut section between the two bison populations. The Shannon index was used to compare diversity levels among sections and between populations. Single-factor ANOVA with the Tukey HSD post-hoc test was used for comparisons among sections, while t-tests with Bonferroni corrections were used for comparisons between populations. Exploratory and multivariate statistics consisted of hierarchical cluster analysis, SIMPER, principal coordinates analysis (PCoA), and PERMANOVA. Relative abundance data were square root transformed, and then used to generate a Bray-Curtis similarity matrix, from which the PCoA ordination plot was produced. These analyses were performed in the PRIMER + PERMANOVA 6 software package [42] and in the R programming language (R [43]).

## Results

**Fig. 1:**
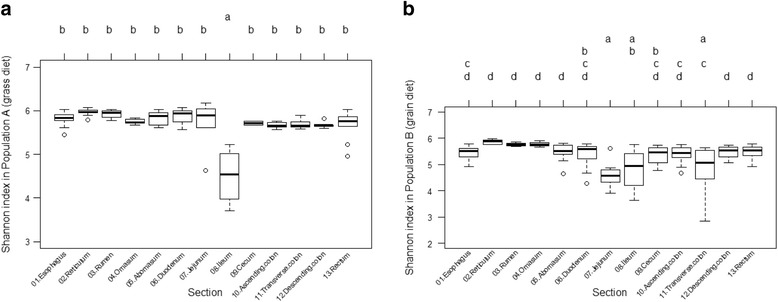
Microbial diversity along the length of the bison digestive tract. Letters above the chart indicate results of Tukey’s HSD test; sections marked with different letters were significantly different from one another. **a** Diversity of the gut microbiota in forage-finished bison, as measured by the Shannon index. **b** Diversity of the gut microbiota in grain-finished bison, as measured by the Shannon index

An average of 37,386 sequences per sample were retained after quality assurance. Samples were rarefied to 1000 sequences per sample, yielding a total of 25,428 OTUs across all samples. In Population A, diversity differed significantly among sections, and was lowest in the ileum (*P* < 0.05). Bison from Population B also exhibited significant differences in diversity among sections (*P* < 0.05), but there was no clear trend (Fig. 1A and B). Population A tended to exhibit significantly higher levels of diversity for a given gut section. Shannon index values were higher for bison from Population A in the esophagus, rumen, and jejunum (*P* < 0.004 in all cases). The three most common OTUs detected were in the phyla *Bacteroidetes* and *Firmicutes*. *Paraprevotellaceae* (Phylum *Bacteroidetes*) was more abundant in the colon among bison from Population A, and was more abundant in the hindgut generally among bison from Population B. *Bacteroidales* (Phylum *Bacteroidetes*) was more abundant in the foregut among bison from both populations. Finally, *Peptostreptococcaceae* (Phylum *Firmicutes*) was more abundant in the ileum and cecum among bison from Population A, but more abundant in the jejunum and ileum among bison from Population B.

**Fig. 2:**
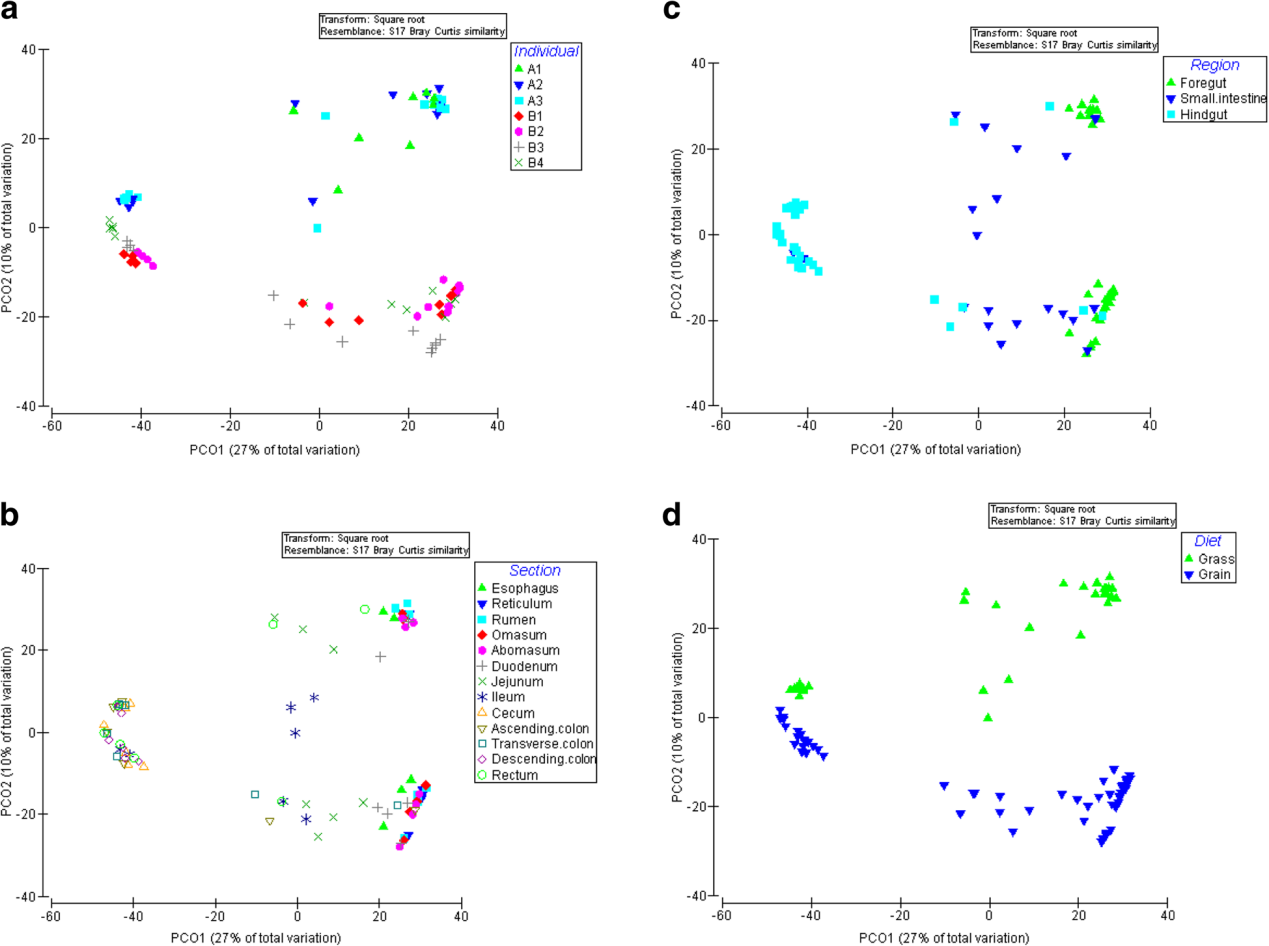
Principal coordinates analysis (PCoA) of microbial operational taxonomic units (OTUs) for entire digestive tract in both populations (forage- and grain-fed) of captive bison. **a** Colors represent the seven bison used in the study. **b** Colors represent the 13 sections of the digestive tract. **c** Colors represent the three regions of the digestive tract. D) Colors represent the two bison populations sampled, each with a different diet

In each population, microbial community composition varied significantly by gut section, as indicated by PERMANOVA (*P* < 0.05), although not typically within the same gut region (Figs. 2A–2D). Thus, for a given diet, the three main fermentation chambers of the foregut (reticulum, rumen, and omasum) had similar microbial communities, as did the five main sections of the hindgut (cecum, ascending colon, transverse colon, descending colon, and rectum). Population A also exhibited similarity in community composition between sections from two different regions of the digestive tract, namely the abomasum and duodenum (*P* = 0.073), and the jejunum and rectum (*P* = 0.161). No such extra-regional similarities were detected in Population B. *Bacteroidetes* were at their lowest relative abundance in the small intestine, specifically the ileum of bison from Population A (*P* < 0.001), and in the duodenum, jejunum, and ileum of bison from Population B (*P* < 0.001). In contrast, *Firmicutes* had their highest relative abundance in the small intestine, namely the ileum of bison from Population A (*P* < 0.0001), and the jejunum and ileum of bison from Population B (*P* < 0.001). *Proteobacteria* and *Tenericutes*, although the third and fourth most abundant phyla, respectively, exhibited no clear trend across the digestive tract in either population (*P* > 0.05).

**Fig. 3:**
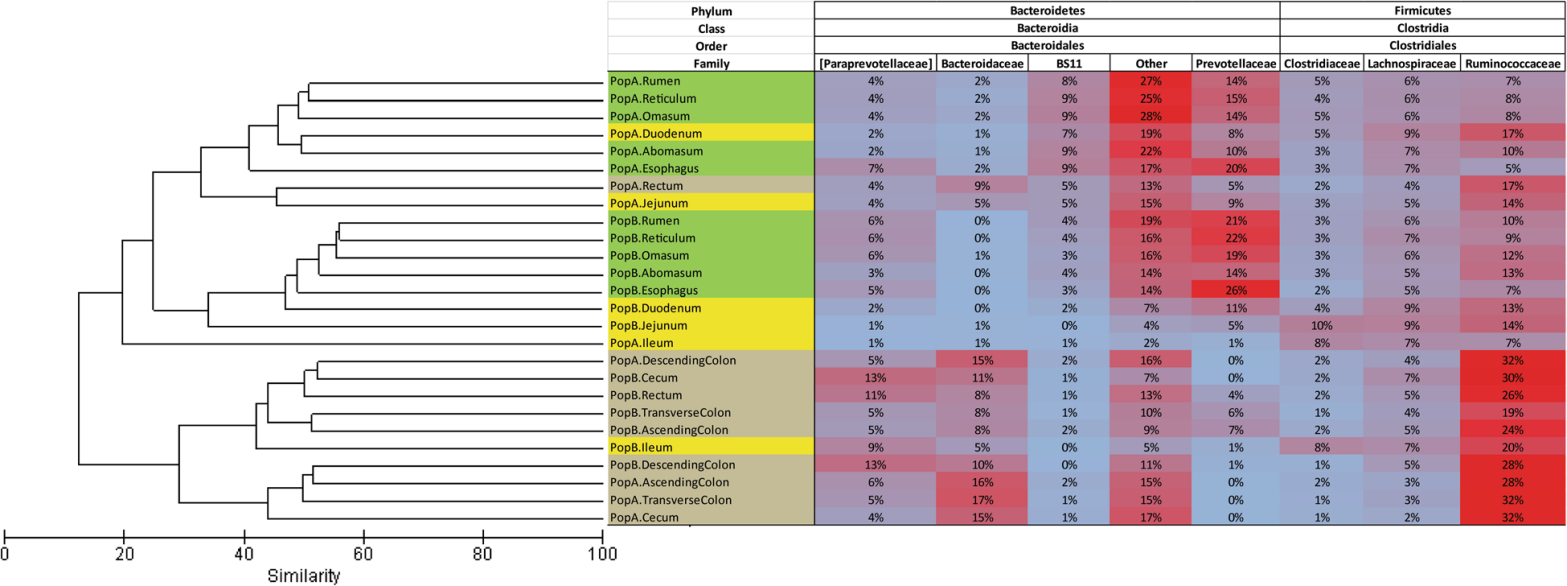
Heat map showing bacterial families with a mean relative abundance of at least 3% across all samples, paired with hierarchical cluster analysis of Bray-Curtis similarity among digestive tract sections in forage-finished (Population A) and grain-finished (Population B) bison. These families represent 27–77% of the microbial community in each gut section

**Fig. 4:**
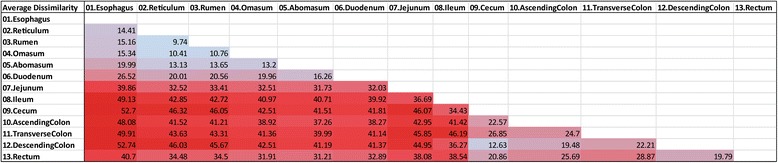
Results of SIMPER analysis, showing average percent dissimilarity among the 13 sections of the digestive tract. Brighter tones of red represent higher levels of dissimilarity, while brighter tones of blue represent lower levels of dissimilarity

Gut section had a greater effect on microbial community composition than population. Thus, foregut communities between the two populations were more similar to each other than they were to hindgut communities from the same population. Likewise, hindgut communities resembled each other more than either resembled corresponding foregut communities (Fig. 3). This finding was corroborated by SIMPER analysis (Fig. 4), which indicated that the reticulum, rumen, and omasum were more similar to one another in the foregut (average dissimilarity <11%), while the cecum and descending colon were similar in the hindgut (average dissimilarity <13%).

**Fig. 5:**
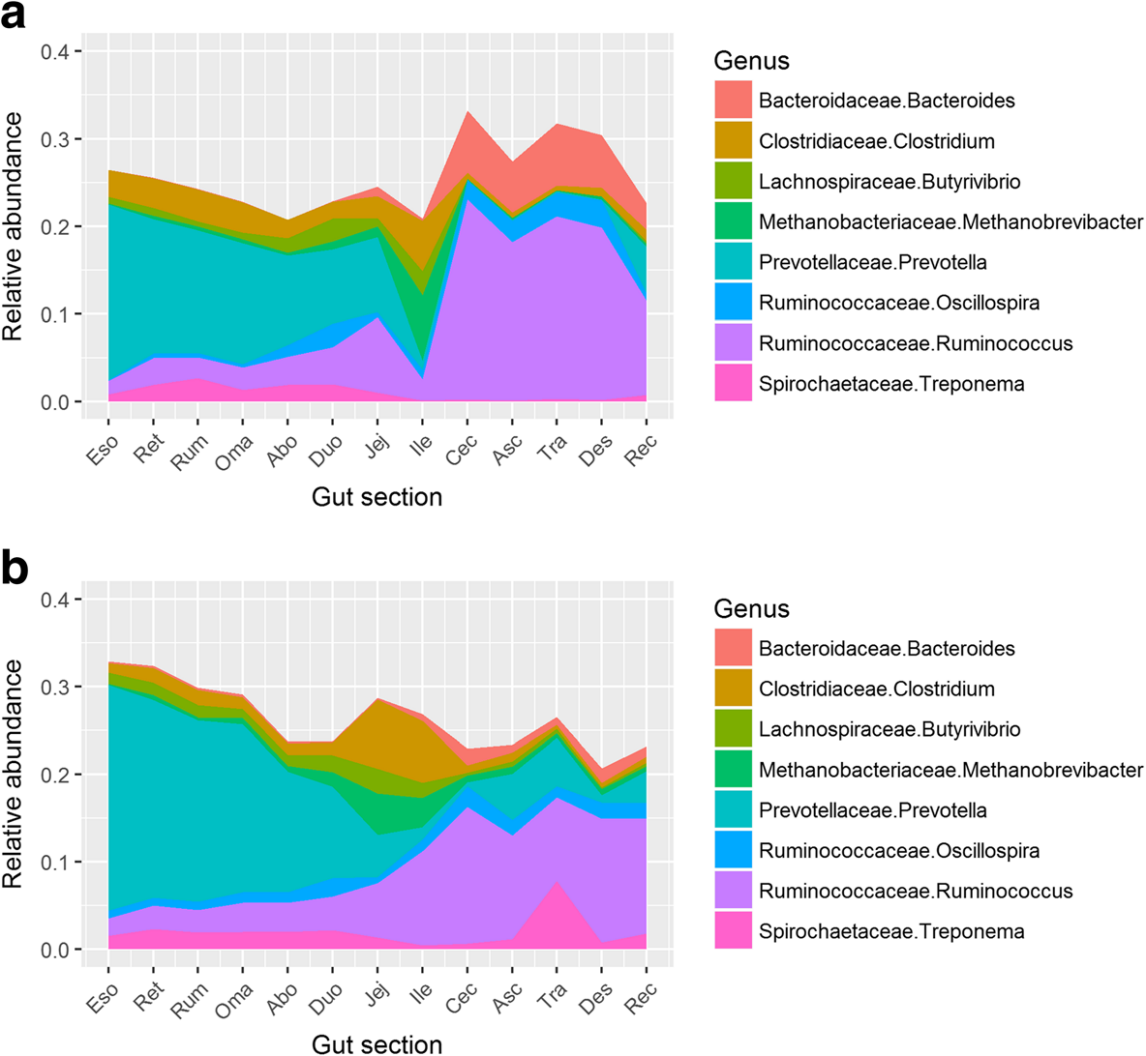
Stacked area plots showing the eight most abundant genera on average in each of the 13 sections of the digestive tract. **a** Bison from Population A, which were grass-finished. **b** Bison from Population B, which were grain-finished. Eso = Esophagus, Ret = Reticulum, Rum = Rumen, Oma = Omasum, Abo = Abomasum, Duo = Duodenum, Jej = Jejunum, Ile = Ileum, Cec = Cecum, Asc = Ascending colon, Tra = Transverse colon, Des = Descending colon, and Rec = Rectum

However, for a given section, there was a significant effect of population as well, as indicated by PERMANOVA (*P* < 0.05). T-tests were used to compare bacterial relative abundance at a given taxonomic level between populations for each digestive tract section. Here sections with significantly different relative abundances of bacteria are reported. Bacteria in the phylum *Bacteroidetes* were more abundant for Population A in the jejunum (*P* < 0.001) and ascending colon (*P* < 0.001). Bacteria in the phylum *Firmicutes* were more abundant for Population B in the jejunum (*P* < 0.001) and rectum (*P* = 0.002). Bacteria in the phylum *Proteobacteria* were more abundant for Population B in the omasum (*P* = 0.003), and those in Class *Gammaproteobacteria* were more abundant for Population B in the reticulum (*P* = 0.001), abomasum (*P* < 0.001), duodenum (*P* = 0.007), and descending colon (*P* = 0.003). Overall, *Ruminococcus* (Phylum *Firmicutes*) exhibited a higher relative abundance in the hindgut of bison from Population A, while *Prevotella* (phylum *Bacteroidetes*) exhibited a higher relative abundance in the foregut of bison from Population B (Fig. 5).

## Discussion

In each population, microbial community composition was significantly different among gut sections from different regions, but similar among those within the same region, namely the multi-chambered stomach and large intestine. This is probably because, although anatomically partitioned, each of these two regions probably functions as a unit [44] The present study on bison, like previous work on cattle [44, 45], sheep [46], and moose [27, 47] also shows that the microbial communities of the rumen and colon are distinct. Thus, while fecal samples can be used as noninvasive colonic samples, they do not represent ruminal communities. Further work is needed to determine if microbial taxa or their ratios in fecal samples may be used as indicators of these or other taxa in the rumen.

For both bison populations studied, diversity as indicated by the Shannon index appears to be lowest in the small intestine, and for bison from Population A (forage-finished), diversity was lowest in the ileum specifically, although the other sections showed no clear trend. The two dominant bacterial phyla in the digestive tract were the *Bacteroidetes* and *Firmicutes*, as is the case in all known mammals [48–51]). *Bacteroidetes* exhibited their lowest relative abundance in the small intestine, while *Firmicutes* reached their highest relative abundance there.

Earlier research has shown that, despite high resource availability, the small intestine tends to harbor the lowest levels of microbial biomass and diversity of any gastrointestinal section, for two main reasons [52]. The first is that digesta have a relatively short retention time in the small intestine due to peristaltic movement, which gives microbes less time to proliferate there [53]. The second is the influence of the section just anterior to the small intestine, the abomasum (true stomach). While the pH of most gut sections ranges from 5 to 7, the abomasum has pH levels of 2–4, the lowest of the ruminant digestive tract [6, 54, 55]. Although the small intestine receives this gastric acid and begins buffering it, that takes time. Digesta pass through the long ruminant small intestine, which is about 20 times the length of the animal, or some 40 m long in bison and cattle. During this time, pH remains relatively low in the duodenum and jejunum, and does not return to near neutral levels until the ileum. In addition to acid, the small intestine also receives bile from the gall bladder and enzymatic secretions from the pancreas. Together, these three inputs create a relatively harsh environment for most microbes [53]. In this environment, the Gram- *Bacteroidetes* could be at a disadvantage compared to the *Firmicutes*, which have a thick peptidoglycan Gram + cell wall [56].

The effect of gut region on community composition was stronger than that of population, as microbial communities were more similar in the same region between populations, than to another region within the same population (Fig. 3). Nevertheless, the bison from Population A (forage-finished) appeared to support greater microbial diversity in most sections of the digestive tract, except the ileum. Previous work with captive domestic cattle has shown that age, sex, and location have less of an effect on microbial community composition than diet [25, 57], although the ruminal microbiota of cattle have been observed to vary with both age and diet [58], and those of moose (*Alces alces*) with age, weight, and location, but not sex [27, 47]. Like other sexually dimorphic ungulates, bison exhibit sexual segregation for most of the year [59]. Accordingly, isotopic and chemical analyses have shown that bison diets differ with age and sex [60, 61] although chloroplast gene sequence-based research has shown that the bison diet does not vary with age or sex [62]. Differences in age, sex, and location between bison populations in the present study make interpretation of the results more difficult, but dietary differences could have had a greater effect on gut community composition than these other factors. Thus, differences in community composition between these populations for a given gut section may be due primarily to forage-finishing in Population A and grain-finishing in Population B. Specifically, the present study suggests that a forage-based diet may be associated with overall greater gut microbial diversity in bison (Fig. 1). This finding is consistent with those of other studies, which have shown that fiber-based diets promote higher levels of microbial diversity than finishing diets, because the fermentation of fiber generates more byproducts than that of starch [63, 64].In many gut sections, *Bacteroidetes* were more abundant with a forage diet, while *Firmicutes* were more abundant with a grain diet. This mirrors what has been found in studies on obesity in humans and mice, where a more natural, less calorific diet that is rich in protein and fiber seems to favor *Bacteroidetes*, while an artificial, energy-rich diet of starch and fats seems to favor *Firmicutes* [22, 65, 66]; [67, 68]. Thus, the gut microbiota of grain-fed bison appears to resemble those of other animals fed a diet high in starchy, processed foods. At the phylum level, *Proteobacteria* were more abundant with a grain diet only in the omasum. Moreover, at the class level, a higher relative abundance of *Gammaproteobacteria* was associated with the grain diet throughout much of the digestive tract.

## Conclusions

Within a given population, microbial community composition differed among sections of the digestive tract, but was similar for sections within the same region, especially the foregut and hindgut. The sections of the small intestine or midgut were overall similar to one another, but exhibited some similarities to the other regions as well. Shannon diversity was lowest in the small intestine, likely because of a generally short retention time in the small intestine, and the influence of low pH from the stomach, bile from the gall bladder, and digestive enzymes from the pancreas. This may be why *Firmicutes*, with their thick cell wall, dominate in the small intestine. The present study also found microbial community compositional differences for a given gut section from two bison populations, possibly due to differences in diet (forage- vs. grain-fed). However, microbial community composition was more divergent among regions of the gut than between dietary groups, indicating that physiological conditions along the digestive tract play a larger role in structing microbial communities than does diet.

It is noteworthy that certain bacterial groups exhibited differences in relative abundance between the rectum and elsewhere in the GI tract. Thus, although fecal sampling is convenient and noninvasive, one must be cautious when extrapolating abundances of bacteria in fecal samples to elsewhere in the alimentary canal [44]. Given previous research on grain-fed cattle and bison, as well as on obese mice and humans, it is likely that the higher levels of *Firmicutes* and *Gammaproteobacteria* found in the grain-finished bison represented dysbiosis [69, 70]; [71]. However, the bison in this study were not assessed for ruminal acidosis, anorexia, or the shedding of enterohemorrhagic bacteria to their environment. Additional research is needed to evaluate the clinical relevance of symbiotic communities brought about by artificial feeding methods in bison.
